# Evaluation of the dosimetric effect of scattered protons in clinical practice in passive scattering proton therapy

**DOI:** 10.1002/acm2.13284

**Published:** 2021-05-25

**Authors:** Chankyu Kim, Yeon‐Joo Kim, Nuri Lee, Sang Hee Ahn, Kwang Hyeon Kim, Haksoo Kim, Dongho Shin, Young Kyung Lim, Jong Hwi Jeong, Dae Yong Kim, Wook‐Geun Shin, Chul Hee Min, Se Byeong Lee

**Affiliations:** ^1^ Proton Therapy Center National Cancer Center Korea Gyeonggi‐do Republic of Korea; ^2^ Department of Radiation Oncology National Medical Center Seoul Republic of Korea; ^3^ Department of Radiation Oncology Yonsei Cancer Center Yonsei University College of Medicine Seoul Republic of Korea; ^4^ Department of Neurosurgery Inje University Ilsan Paik Hospital Gyeonggi‐do Republic of Korea; ^5^ Department of Radiation Oncology Seoul National University Hospital Seoul Republic of Korea; ^6^ Department of Radiation Convergence Engineering Yonsei University Gangwon‐do Republic of Korea

**Keywords:** proton therapy, passive scattering, scattered protons, aperture, snout, range compensator

## Abstract

The present study verified and evaluated the dosimetric effects of protons scattered from a snout and an aperture in clinical practice, when a range compensator was included. The dose distribution calculated by a treatment planning system (TPS) was compared with the measured dose distribution and the dose distribution calculated by Monte Carlo simulation at several depths. The difference between the measured and calculated results was analyzed using Monte Carlo simulation with filtration of scattering in the snout and aperture. The dependence of the effects of scattered protons on snout size, beam range, and minimum thickness of the range compensator was also investigated using the Monte Carlo simulation. The simulated and measured results showed that the additional dose compared with the results calculated by the TPS at shallow depths was mainly due to protons scattered by the snout and aperture. This additional dose was filtered by the structure of the range compensator so that it was observed under the thin region of the range compensator. The maximum difference was measured at a depth of 16 mm (8.25%), with the difference decreasing with depth. Analysis of protons contributing to the additional dose showed that the contribution of protons scattered from the snout was greater than that of protons scattered from the aperture when a narrow snout was used. In the Monte Carlo simulation, this effect of scattered protons was reduced when wider snouts and longer‐range proton beams were used. This effect was also reduced when thicker range compensator bases were used, even with a narrow snout. This study verified the effect of scattered protons even when a range compensator was included and emphasized the importance of snout‐scattered protons when a narrow snout is used for small fields. It indicated that this additional dose can be reduced by wider snouts, longer range proton beams, and thicker range compensator bases. These results provide a better understanding of the additional dose from scattered protons in clinical practice.

## 1 INTRODUCTION

Charged particles, including protons and carbon ions, are being increasingly introduced into modern radiation therapy. The physical and biological characteristics of proton beams, including a low entrance dose and the absence of an exit dose, make proton beams more attractive than conventional photon beams in radiation therapy.^1^ At present, more than 80 proton therapy facilities are in operation worldwide, with the number increasing every year.^2^

Despite the clinical use of proton therapy for many years, several uncertainties remain. Conventional treatment planning systems (TPS) have limitations in dose calculation for proton therapy. Uncertainties in dose calculation may be caused by, for example, conversion of Hounsfield units (HU) to relative proton stopping power, limited modeling of multiple Coulomb scattering, biological effects, and scattered and secondary radiation from the treatment nozzle.^3^, ^4^, ^5^, ^6^

Due to the inherent limitations of conventional pencil‐beam algorithms, scattered protons from a field‐limiting aperture or collimator have not been fully included in dose calculation by commercial TPSs for passive scattering proton therapy.^7^, ^8^ Previous studies focused on the dosimetric influence of the scattered protons from the aperture, which is mounted onto the end of the nozzle to shape the proton beam onto the target, because it is likely the most significant contributor to proton scattering.

The influence of scattered protons from slit collimators in small proton fields between 2 mm and 20 mm was investigated by Monte Carlo simulation for a proton beam of 150 MeV.^9^ These simulations showed that the contribution of collimator scattered protons was not negligible, constituting 20% of the total dose at the exit of the collimator and 5% of the total dose even 15 cm away from the collimator. This influence of scattered protons from the edge of the collimator aperture was also detected in a series of studies of low‐energy proton beams used in ocular proton therapy.^10^, ^11^, ^12^ The size of the final collimator aperture was a little larger (24 mm) than in the previous study, and the measured and simulated depth‐dose profiles showed the contribution of scattered protons at shallow depths. Following these studies, emphasizing the dosimetric influence in small proton fields, Titt et al^13^ investigated that the dependency of the dosimetric impact of collimator‐scattered protons on several variables, including proton beam range, the modulation width of the spread‐out Bragg peak (SOBP), field size, and the air‐gap between the collimator and the phantom.

Investigations of the effects of aperture‐scattered protons led to the development of extended algorithms that included this scattering from the aperture to more precisely calculate patient dose.^12^, ^14^, ^15^, ^16^ In addition, new aperture structures that produce fewer scattered protons were developed.^17^, ^18^ But despite these attention to aperture‐scattered protons, they are not considered in commercial TPSs. The dosimetric effects in clinical practice, when the range compensator is included, have not been reported.^13^

The present study was designed to evaluate the potential dosimetric effects of scattered protons from field‐limiting apertures in clinical practice, including the range compensator in passive scattering proton therapy. By comparing the measured dose with doses calculated by TPS and Monte Carlo simulation, the effect of the scattered protons could be determined, even after these protons passed through the range compensator. The filtration of this effect by the structure of the range compensator was investigated, and the contribution of a snout and an aperture to the dose of scattered protons was analyzed by Monte Caro simulation. In addition, the effects of snout size, beam range, and thickness of the range compensator base on the dosimetric effect of scattered protons were evaluated.

## 2 MATERIALS AND METHODS

### 2.A Monte Carlo simulation of the proton treatment nozzle

**Fig. 1:**
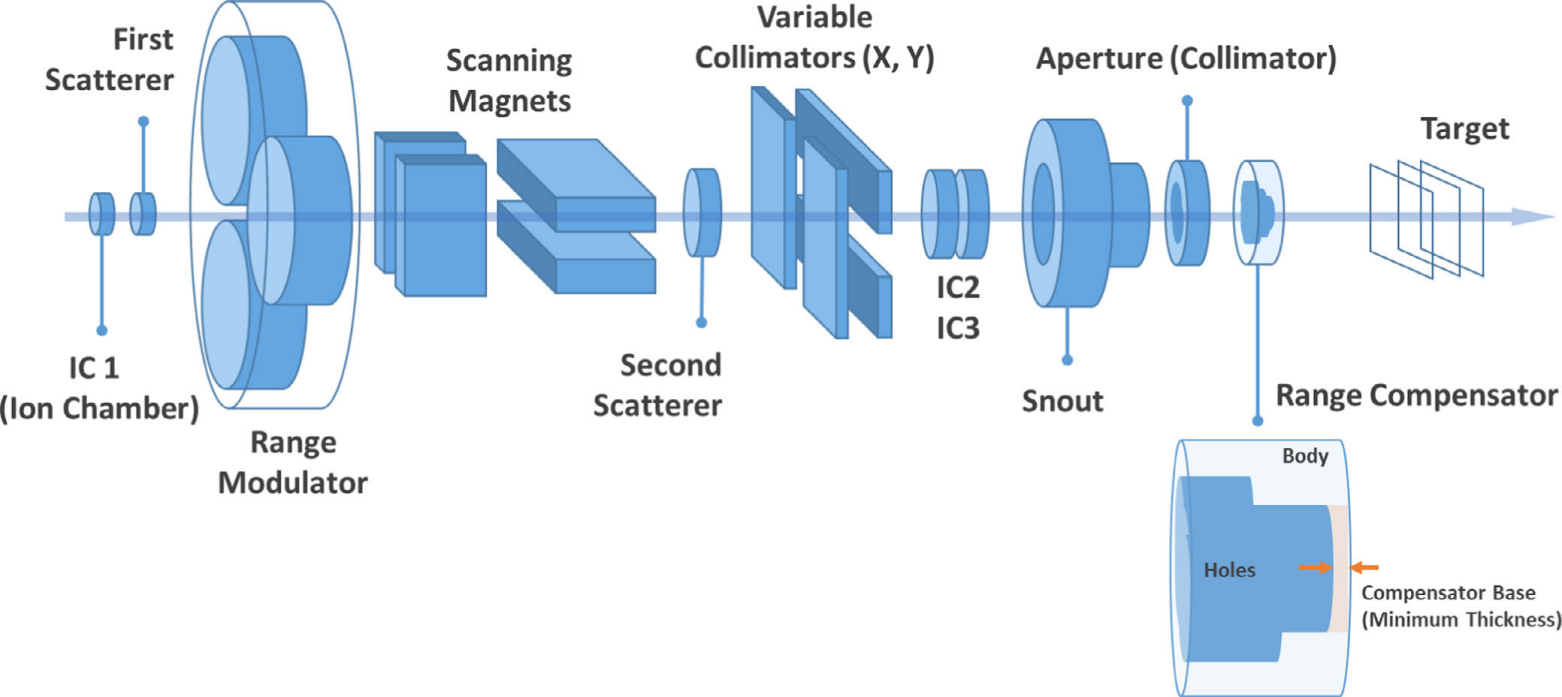
Diagram of the proton therapy nozzle in double‐scattering delivery mode and the structure of a range compensator.

A Monte Carlo simulation system of a proton therapy nozzle in double‐scattering delivery mode at the National Cancer Center Korea (Fig. 1) was developed for independent dose verification of treatment plans.^19^ The design of the model nozzle was based on the manufacturer’s blueprints (the IBA universal nozzle, IBA) and a previously designed model based on Geant4.^20^, ^21^ The tool for particle simulation (TOPAS, 3.1.p03 version)^22^ was used for nozzle modeling and simulation of proton transport. In the previous study,^19^ the developed Monte Carlo simulation model of the scattering proton therapy nozzle was validated by comparison of its simulated percent depth–dose (PDD) profiles with previously measured PDD profiles. In addition, the results calculated by the Monte Carlo simulation and TPS for a lung phantom, an internal mammary node, and an abdomen were compared to verify the feasibility of the developed system in clinical practice.^19^ The developed Monte Carlo model showed good agreement with both the actual measurement and TPS calculation, differing less than 1 mm in range and modulation width.^19^

In this study, the dose distribution in a uniform water phantom was calculated using the Monte Carlo simulation system. Virtual treatment plans and their beam parameters, which were determined by the converting algorithm (Convalgo, IBA), were imported into the simulation system to set up the beam nozzle. In the simulation, protons were assumed to be emitted from the beam entrance with the determined mono‐energy and passed through the preset nozzle structures. The energy, positional, and angular spread of the initial proton beam were assumed as a single Gaussian distribution with parameters benchmarked by measurements performed in the previous study.^21^ The apertures and range compensators in the simulation were modeled on the designs included in the imported DICOM files and were composed of brass and poly(methyl methacrylate) (PMMA) (Table 1).^19^ The phantom was placed at the same distance from the range compensators as the air gaps in the virtual plans. The material of the phantom was assigned as G4_Water. The other simulation parameters were the same as those in the reference.^19^ The simulation parameters are briefly listed in Table 2.

**Table 1:** Characteristics of the brass and PMMA used in the simulations.

Material	Density (g/cm^3^)	Elements	Weight fraction
Brass	8.32	Copper	0.5775
		Lead	0.0345
		Iron	0.0025
		Aluminum	0.0034
		Zinc	0.3821
PMMA	1.18	Hydrogen	0.0810
		Carbon	0.5998
		Oxygen	0.3192

**Table 2:** A summary of parameters used in the simulation.

Category	Parameter	Value
Initial proton beam	Type	Beam
	Particle	Proton
	Beam position distribution model	Gaussian
	Beam position spread x/y	0.1 cm
	Beam angular distribution model	Gaussian
	Beam angular spread x/y	0.01 deg
	Beam energy	Varied depending on the plan
	Beam energy spread	Varied depending on the energy
Physics	Physics modules (The default physics list in TOPAS)	G4EM‐Standard_opt4
		G4h‐QGSP_BIC_HP
		G4Decay
		G4Ion‐Binarycascade
		G4h‐Elastic
		G4Stopping
Nozzle composition	The first and second scatterer	Varied depending on the plan
	Range modulation wheel	
	Snout type and position	
Patient‐specific components	Aperture material	Blass (Table 1)
	Range compensator material	PMMA (Table 2)
Phantom	Position from the range compensator	Air gap in the plan
	Material	G4_Water
	Voxel size	1.25 mm
Simulation runs	Sum of the number of histories in run (Particles in each run = beam weight * input value)	> 10^9^ particles
The other parameters	Default values in TOPAS^23^	

To ensure sufficient statistical accuracy, each simulation included more than 10^9^ primary protons. The secondary neutrons were not included in the simulation. The calculated doses in the phantoms were recorded as DICOM files, each of which consists of 256 × 256 × 256 cubic voxels with edge lengths of 1.25 mm. The DICOM files from the simulation were imported into the TPS and compared with the doses calculated by the TPS. For comparison, the doses calculated from the simulations were normalized to the doses on TPS at the center of the SOBP on PDD profiles.

### 2.B Treatment plans for validation and evaluation

**Fig. 2:**
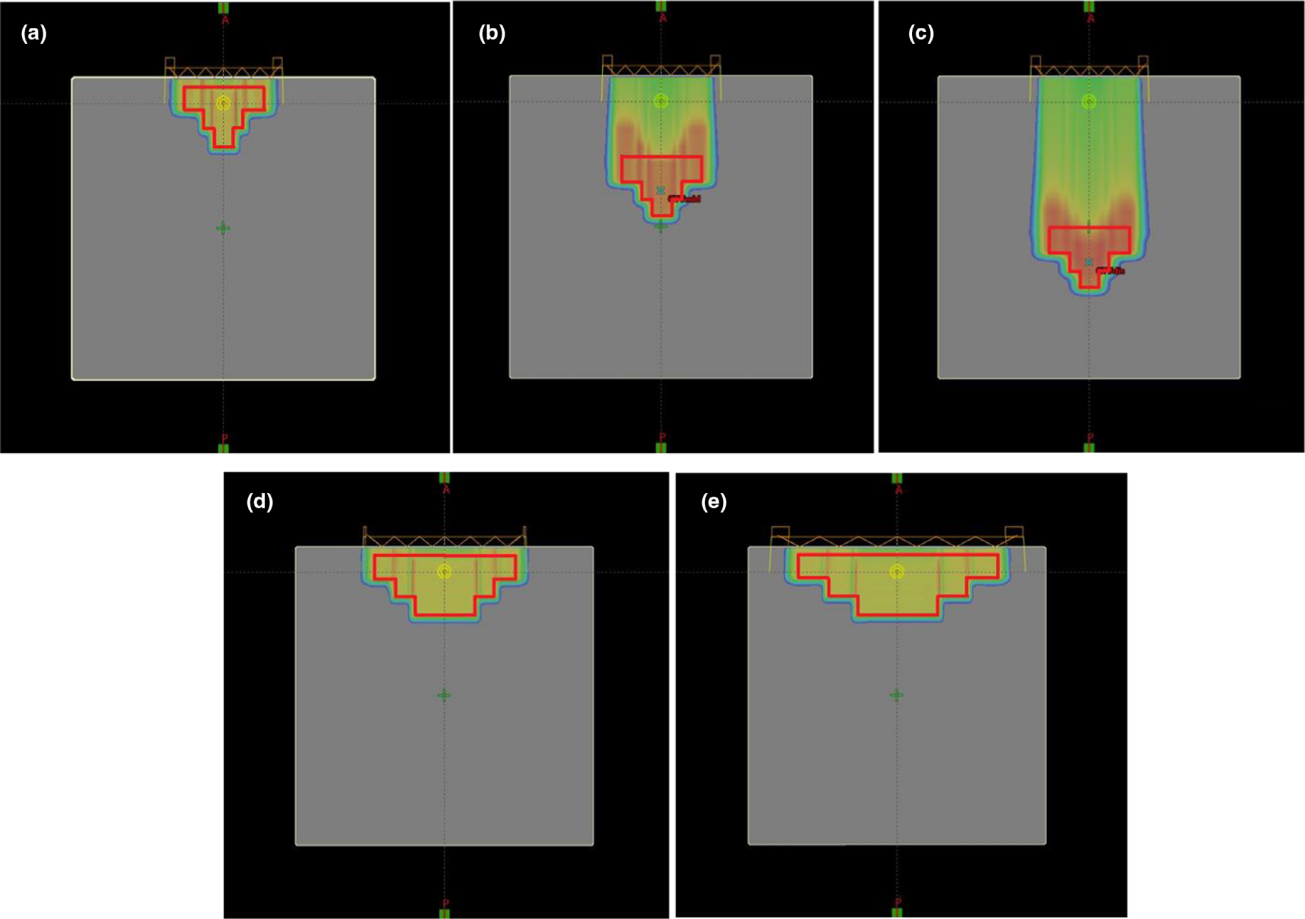
The PTVs and calculated doses in the (a) Proximal 100, (b) Middle 100, (c) Distal 100, (d) Proximal 180, and (e) Proximal 250 virtual treatment plans on TPS. All plans were based on a virtual water phantom measuring 30 cm × 30 cm × 30 cm, with a single AP beam covering the PTVs. The plans were normalized to deliver a single fraction of 200 cGy to the center of the PTVs.

Virtual treatment plans were used to evaluate and analyze the dosimetric influence of scattered protons from the nozzle. These plans utilized a water phantom and were based on the proton convolution superposition (PCS) algorithm in the TPS (Eclipse 13.7, Varian). The planning target volume (PTV) of each plan was in the shape of an overturned three‐tier cake, with the cake structures in different plans having different depths, radii, and positions (Figure. 2). All plans used the anterior‐posterior (AP) beam, with the beam range and modulation width of SOBP chosen to cover the PTV. The details of these PTVs and the beam properties of the plans are shown in Table 3. The aperture and range compensator in each plan were designed to match the shape of the PTV, including considerations of snout sizes, milling compensation (1.2 cm drill bit), smearing margin (0.2 cm), and the range compensator base (minimum thickness, 2 mm). The distance between the range compensator and the solid water phantom (air gap) was set at 8.5 cm. Each plan was normalized to deliver a single dose of 200 cGy to the center of the PTV.

**Table 3:** Details of the PTVs and beam properties of the plans.

Plan	PTV				Snout (inner diameter)	Aperture (inner diameter)	Beamline Properties	
	Tear	Depth (cm)	Radius (cm)	Position			Range (cm)	Modulation width (cm)
Proximal 100	Top	2.3	8	1 cm beneath the surface	SNT 100 (11 cm)	9.9 cm	7.31	6.21
	Middle	1.8	4					
	Bottom	1.8	2					
Middle 100	Top	2.3	8	8 cm beneath the surface	SNT 100 (11 cm)	9.9 cm	14.47	6.19
	Middle	1.8	4					
	Bottom	1.8	2					
Distal 100	Top	2.3	8	15 cm beneath the surface	SNT 100 (11 cm)	9.9 cm	21.53	6.18
	Middle	1.8	4					
	Bottom	1.8	2					
Proximal 180	Top	2.3	14	1 cm beneath the surface	SNT 180 (16 cm)	16 cm	7.40	6.35
	Middle	1.8	10					
	Bottom	1.9	6					
Proximal 250	Top	2.3	20	1 cm beneath the surface	SNT 250 (23 cm)	22.1 cm	7.40	6.35
	Middle	1.8	14					
	Bottom	1.9	8					

### 2.C Verification of the dosimetric effect of the scattered protons

To verify the dosimetric effect of the scattered protons after passage through a range compensator, the delivered dose based on the Proximal 100 treatment plan was measured and compared with the doses calculated by TPS and Monte Carlo simulation. To properly compare with measurements, the verification plan was established on a solid water phantom, which was assigned with a 30 HU value that resulted in a relative proton stopping power of 1.03 on TPS and with a density of 1.0272 g/cm^3^ in the Monte Carlo simulation. The plan was targeted on the same PTV in the Proximal 100 plan (Fig. 2(a) and Table 3) and was normalized to deliver 200 cGy to the center of the PTV in a single fraction. The other parameters, including the beamline properties (AP direction, range 7.31 cm, and SOBP width of 6.21 cm) and air gap (8.5 cm), were the same as those in the original plan (Proximal 100). The aperture and range compensator were identical to those in the original plan.

To assess the effect of scattered protons with depth, the 2‐dimensional dose distribution in the solid water phantom was measured at several depths using the IBA proton therapy machine installed in the second proton treatment room at the National Cancer Center Korea. Apertures and range compensators were manufactured as designed from the verification plan and mounted downstream of the snout. The air gap between the range compensator and the solid water phantom was maintained at 8.5 cm, as in the plan. Therefore, the 2D dose distributions were measured while the air gap was kept constant.

The 2D dose distributions were measured using radiochromic films (Gafchromic EBT3, Ashland Advanced Materials, USA) and an array of ion chambers (MatriXX Evolution, IBA Dosimetry, Germany). The irradiated EBT3 films were scanned with a film scanner (10000XL, EPSON, Japan), and the scanned images were converted to radiation dose using RIT software (RIT classic, Radiological Imaging Technology, USA) with a calibration curve, which was determined using a Farmer‐type ionization chamber (Farmer 30013, PTW, Germany). To compare the measured 2D dose distributions with the 2D dose distributions at each depth in TPS calculation and Monte Carlo simulation, the measured doses were imported into MatriXX operating software (OmniPro‐I’mRT, IBA Dosimetry, Germany). All measured dose distributions were converted and interpolated linearly to 0.5 mm and aligned to match the center. The converted dose distributions were compared in orthogonal profiles and 2D gamma analysis, with the latter used to show dose differences spatially rather than to evaluate the agreement. The criteria for gamma analysis were 2 mm for distance to agreement (DTA) and 3% for dose difference. The same size of the region of interest (ROI) (12 cm × 12 cm) was utilized for comparisons with TPS calculations and simulations at each depth.

### 2.D Dependence of the effect on field sizes and beam ranges

After verifying the dosimetric effects of scattered protons in practice, including a range compensator, the effects of different snout sizes and beam ranges were investigated. The virtual plans with different field sizes and beam ranges (Table 3) were established for this purpose. Differences between the plans were assessed by comparing the results from the Monte Carlo simulation. The additional dose relative to that calculated on the TPS was in the central area that corresponded to the shallow region of the range compensator, and the variation of these additional doses was analyzed with depth.

### 2.E Dependence of the effect on the compensator base

The effect of compensator base thickness on the dosimetric effects of scattered protons was investigated using the virtual Proximal 100 plan in section 2.2. The effects of the three possible compensator bases on virtual treatment plans covering the same target were evaluated. The beamlines were changed to cover the same PTV, and the designs of the aperture and range compensator were changed based on the increased thickness of the range compensator base. The snout position was maintained to keep the same divergence so that the air gap between the compensator and solid water phantom was reduced as the compensator base was increased. The dose distributions were recalculated for range compensators of different base thicknesses in the new virtual plans (Table 4). The results of the treatment plans with 2 mm (Proximal 100 – C2) and 20 mm (Proximal 100 – C20) bases were compared. However, the treatment plan with a 40 mm base was used only for analysis because such a plan is not considered practically applicable due to an increase in the lateral penumbra.

**Table 4:** Details of the treatment plans with different compensator bases.

Plan	PTV	Snout (inner diameter)	Aperture (inner diameter)	Range compensator base (thickness)	Air gap	Beamline Properties	
						Range (cm)	Modulation width (cm)
Proximal 100 ‐ C2	Identical to the Proximal 100 Plan (Table 2)	SNT 100 (11 cm)	9.9 cm	2 mm	8.5 cm	7.31	6.21
Proximal 100 ‐ C20				20 mm	6.7 cm	9.40	6.21
Proximal 100 ‐ C40				40 mm	4.7 cm	11.98	6.35

To verify the effect, the integral depth–dose (IDD) profiles were measured using a multilayer ionization chamber (MLIC) (Zebra, IBA Dosimetry, Germany) at a gantry angle of 270° with the compensators of different bases. Monte Carlo simulation was performed for comparison and analysis of the measured results. Due to lack of the detailed information and complexity of the detector structure, it was difficult to describe the MLIC detector precisely in the simulation. Because the measured results using the MLIC detector showed good agreement with the measured results by the conventional methods using an ionization chamber in a water phantom,^24^ a uniform water phantom was used instead of describing the complex structure of the detector in the simulation. Considering the collecting volume of ion chambers in the MLIC, the dose was scored along the central axis (CAX) in a binned cylinder with 2.5 cm diameter in 1 mm increments. The other parameters for nozzle simulation, including the initial proton distribution and materials, were the same as in the previous simulations.

## 3 RESULTS

### 3.A Verification of the dosimetric effect of scattered protons

**Fig. 3:**
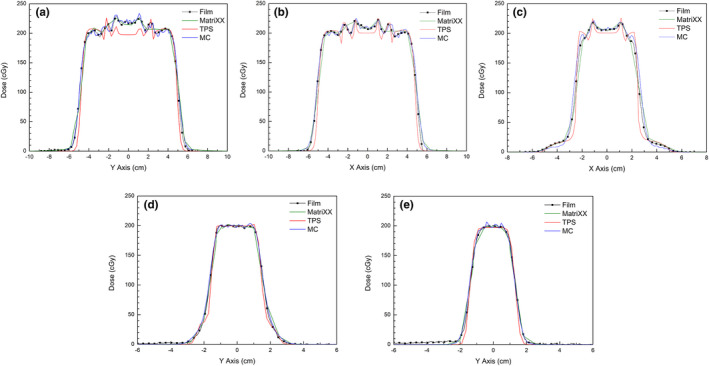
Measured dose profiles (Film, MatriXX) and dose profiles calculated by TPS and Monte Carlo (MC) simulation: (a) on the y‐axis at a depth of 16 mm, (b) on the x‐axis at a depth of 30 mm, (c) on the x‐axis at a depth of 40 mm, (d) on the y‐axis at a depth of 56 mm, and (e) on the y‐axis at a depth of 69 mm.

Comparisons of measured dose profiles and dose profiles calculated by TPS and Monte Carlo simulation on the x‐ and y‐axes at several depths are shown in Figure 3. These dose profiles were normalized to the dose profile measured with MatriXX at a depth of 56 mm, which was expected to be free of the dosimetric effects of scattered protons. The normalization factors calculated at a depth of 56 mm were used for normalizations at other depths. The dose profiles on both the x‐ and y‐axes were symmetric at each depth because the plan was based on covering the symmetric target on CAX. At all depths, the dose profiles calculated by the simulation were in agreement with the dose profiles measured with EBT3 films and MatriXX. In contrast, the calculated profiles by TPS were narrower. These findings indicate that the current PCS algorithm in Eclipse cannot reflect accurately multiple Coulomb scattering, whereas the Monte Carlo simulation does.

Spikes in these profiles were found to occur at the interfaces between pairs of compensator depths and were thought to be caused by rapid changes in compensator depth. Further study is required to explain exactly their source. The spatial resolution of the MatriXX (7.6 mm) was not sufficient to describe this rapid dose change at closer distances, whereas the smaller resolution of the scanned EBT3 film (0.35 mm) was regarded as sufficient. Results from both the TPS calculation and simulation included these spikes, but the spikes observed on the dose profiles from TPS calculations were simpler in shape than the measured dose profiles measured with EBT3 film and the simulated dose profiles. These differences in spikes were caused by the limitations of the TPS calculation of the scattering effect at range compensators.

The most noticeable differences in dose profiles of the TPS calculations were observed in the central region at shallow depths (Fig. 3(a) and 3(b)). In the central region, which corresponds to the thin area of the range compensator, the measured and simulated dose profiles show higher doses than the dose profiles calculated by the TPS. The average differences in doses to the central region between the TPS calculation and the other methods at depths of 16 mm, 30 mm, and 40 mm were 16.5 cGy (8.25%), 7.4 cGy (3.7%), and 4.6 cGy (2.3%), respectively. Compared with the TPS calculation, the additional dose decreased as measurement depth increased, with little difference in dose profiles measured at depths of 56 mm and 69 mm.

**Fig. 4:**
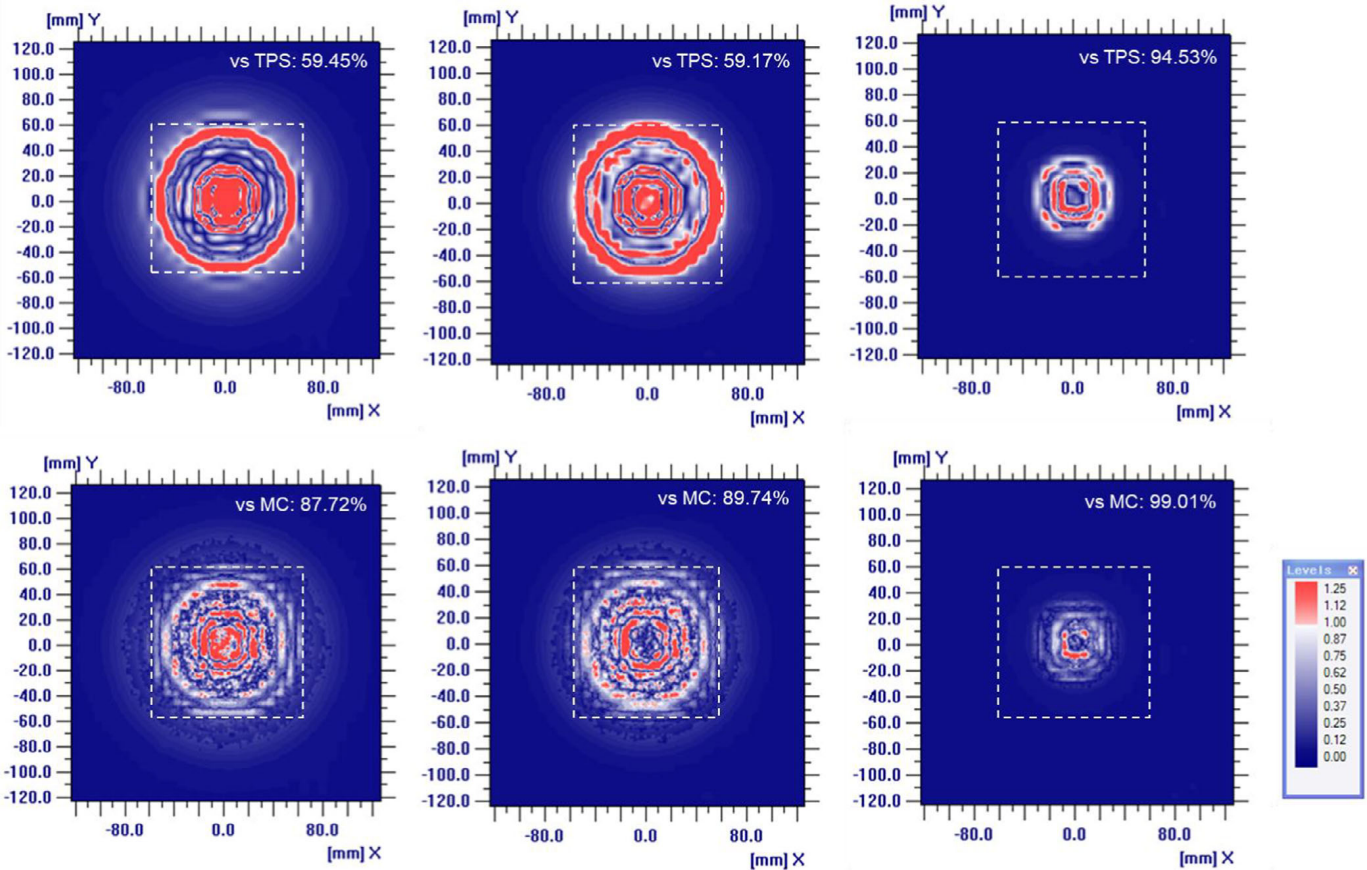
Two‐dimensional gamma analysis of dose distributions measured with a MatriXX and compared with dose distributions calculated by the TPS (above) and Monte Carlo simulation (bottom) at depths of (a) 16 mm, (b) 30 mm, and (c) 56 mm. The criteria for gamma analysis were 3% for dose difference and 2 mm for DTA.

The 2D gamma analysis clearly showed the dose differences in the central region at shallow depths. Figure 4 shows the results of gamma analysis of dose distributions measured with the MatriXX and compared with the dose distributions calculated by the TPS and simulation. Despite the limited spatial resolution of the MatriXX and strict criteria (3% / 2 mm), the gamma analysis between the measured and simulated dose distributions showed acceptable agreement close to 90% passing rate. In contrast, the gamma analysis comparing TPS calculations and measurements at depths of 16 mm and 30 mm showed two red zones, one at the center and the other at the boundary, indicating disagreement. The discrepancy at the boundary was likely due to the difference in the lateral penumbra. The discrepancy at the center was noticeable, but was not observed in the gamma analysis between the measured and simulation results.

**Fig. 5:**
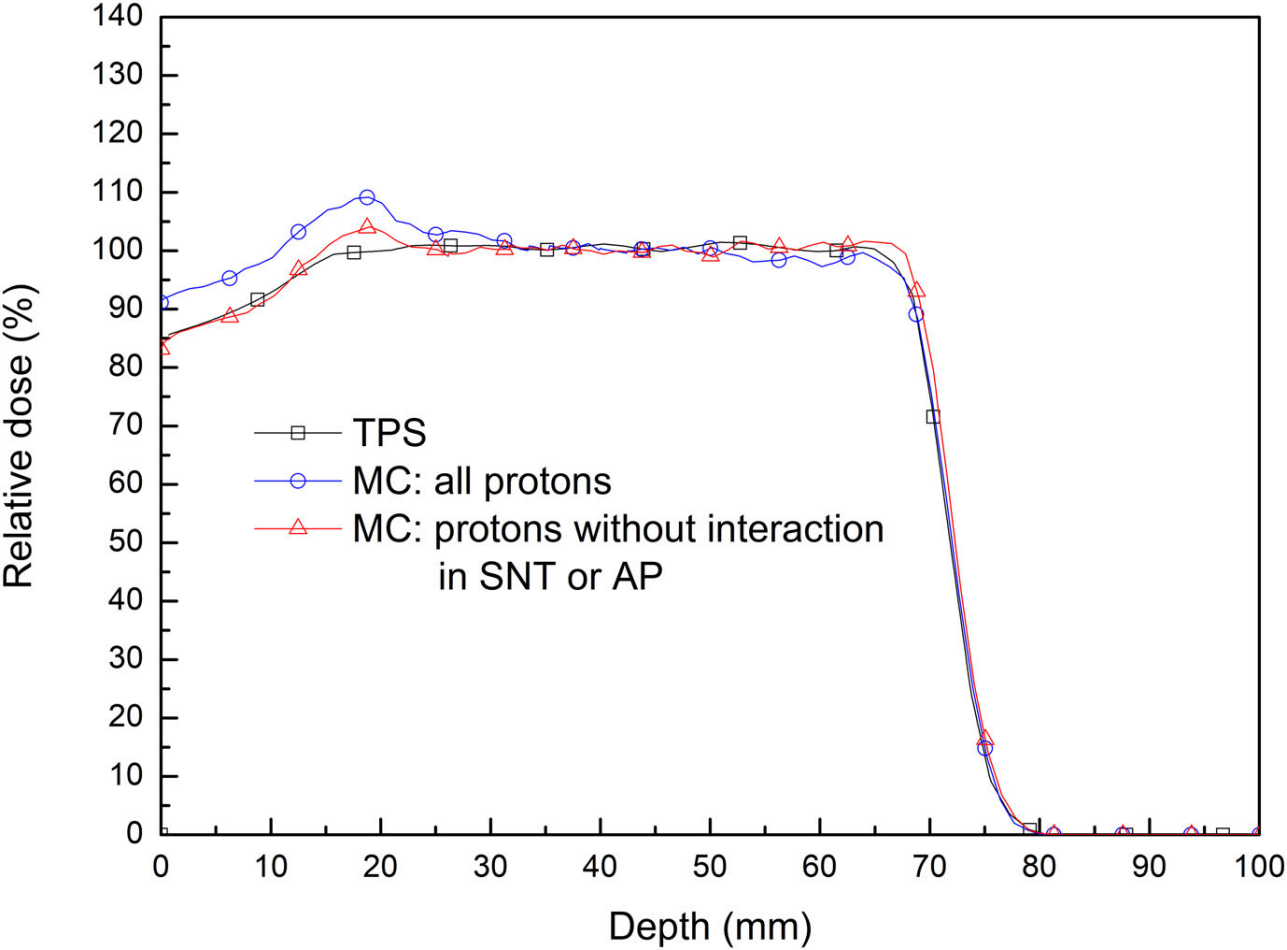
Comparison of the PDD profile calculated by the TPS to the profiles calculated by Monte Carlo (MC) simulation for all protons and protons that did not interact in the snout (SNT) or aperture (AP).

These unexpected additional doses in the central region at shallow depths were likely caused by protons scattered from the snout or aperture. Figure 5 shows a comparison of the calculated PDD profiles on TPS with the profiles from Monte Carlo simulation. All profiles were acquired on the CAX and normalized to the dose at the center of the SOBP. Whereas the TPS calculation showed the profile of the ideal SOBP, the simulated PDD profiles included additional doses at depths ≤40 mm. Similar to the measured results, these additional doses were observed at shallower depths but decreased as depths increased. Exclusion of scattered protons by the filtration of the interacting protons in the snout or aperture in the simulation resulted in a simulated PDD profile approaching the calculated profile on TPS. This difference between profiles resulting from the application of the filter to scoring in the simulation indicated that the observed dose difference in the central region was caused by the scattered protons in the snout or aperture.

**Fig. 6:**
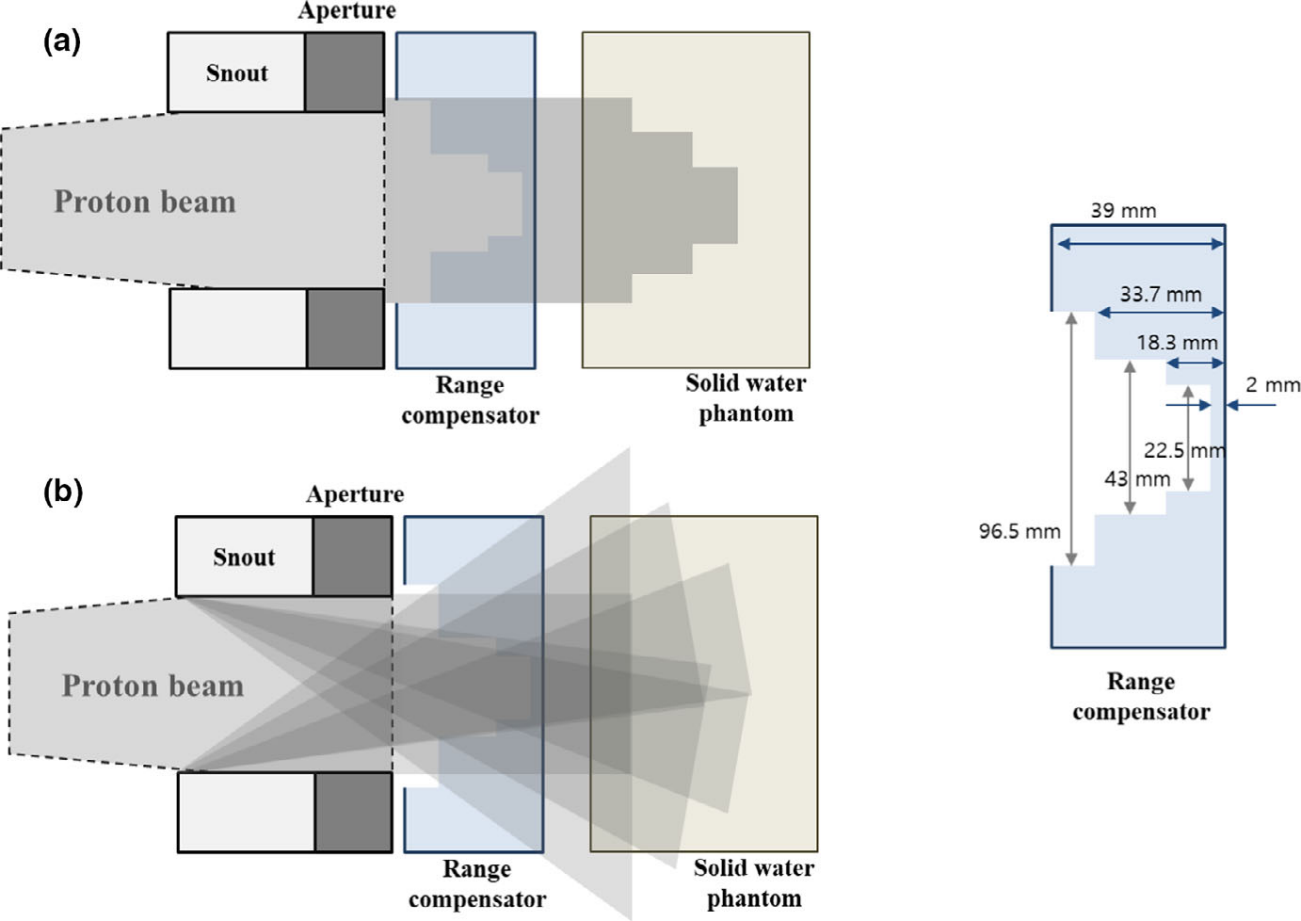
Simplified diagram of dose accumulation by (a) primary protons and (b) protons scattered from the snout or aperture, and considering the range compensator in the virtual treatment plan.

Accumulation of the dose by scattered protons in the central region at shallow depths can be understood by considering the effect of a range compensator (Fig. 6). In the passive scattering proton therapy, protons entering the nozzle are scattered on the beam pathway at various angles, and some of these protons can reach the snout or aperture. Protons scattered by the snout or aperture lose direction, allowing them to pass through the range compensator in directions different from the original direction of primary protons.

The designed range compensator in the virtual plan has different thicknesses from 2 mm to 39 mm. Scattered protons that pass through thick regions of the range compensator lose energy and are therefore eventually absorbed by the superficial region. The only scattered protons that pass through the thin central region of the range compensator can have an effect deep within a phantom. Overlapping of the effects of all scattered protons results in a significant dosimetric effect at the center, whereas the effect on the outside is spread out and not significant. This may explain why an additional dose deposited by scattered protons is observed at the center at shallow depths in the previous measurements.

### 3.B Analysis on the dosimetric effect of scattered protons

**Fig. 7:**
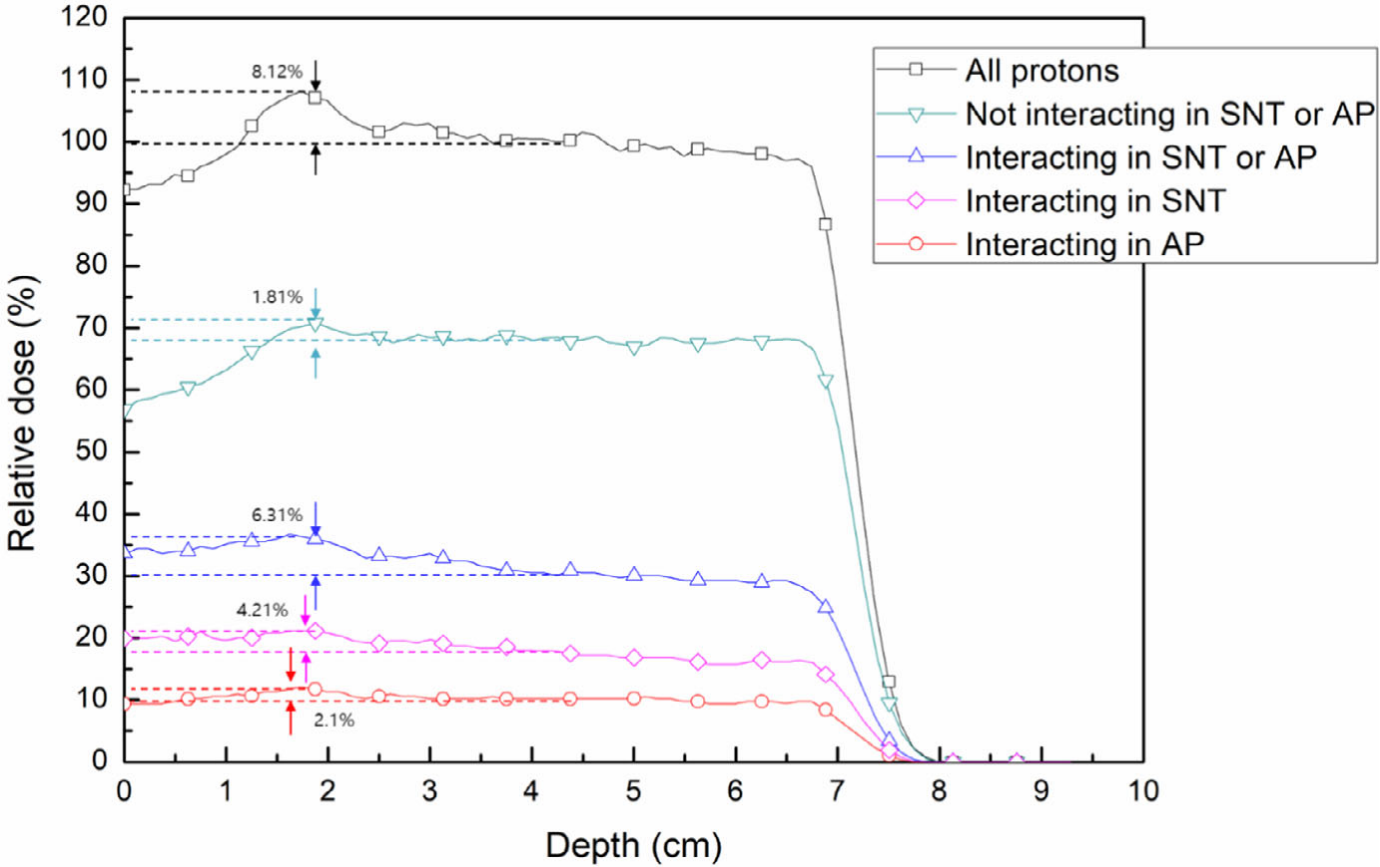
PDD profiles on the CAX with following filters applied to scoring the dose depending on interactions in the snout (SNT) and/or aperture (AP).

The additional dose delivered by scattered protons, which is observed in measurements and Monte Carlo simulations, was analyzed quantitatively in the simulations. The application of filters to scoring in the simulation resulted in the subdivision of the scored dose in the solid phantom into five classes: 1) by all protons, 2) by protons that do not interact in the snout and aperture, 3) by protons that interact in the snout or aperture, 4) by protons that interact only in the snout, and 5) by protons that interact only in the aperture. Figure 7 shows the PDD profiles on the CAX with the applied filters. All scored doses were normalized to the dose at the center of the SOBP of the original simulation, which included all protons. While the dose at the proximal end of the SOBP was 8.12% higher than at the center of the SOBP when all protons were included in the simulation, the dose was only 1.81% higher at the proximal end than at the center when only primary protons which do not interact in the snout or aperture were included.

In contrast, the PDD profile with the other classes of protons, which interact in the snout or aperture, resulted in 6.31% higher additional doses. A comparison of PDD profiles of protons interacting only in the snout and protons interacting only in the aperture showed that the contribution of snout‐scattered protons to the additional dose (4.21%) was almost twice as high as the contribution of aperture‐scattered protons (2.1%). This was reasonable, as the snout (60 cm) was much longer than the aperture (6.5 cm). Taken together, these findings indicate that the snout, not the aperture, is the major source of scattered protons in this plan.

**Fig. 8:**
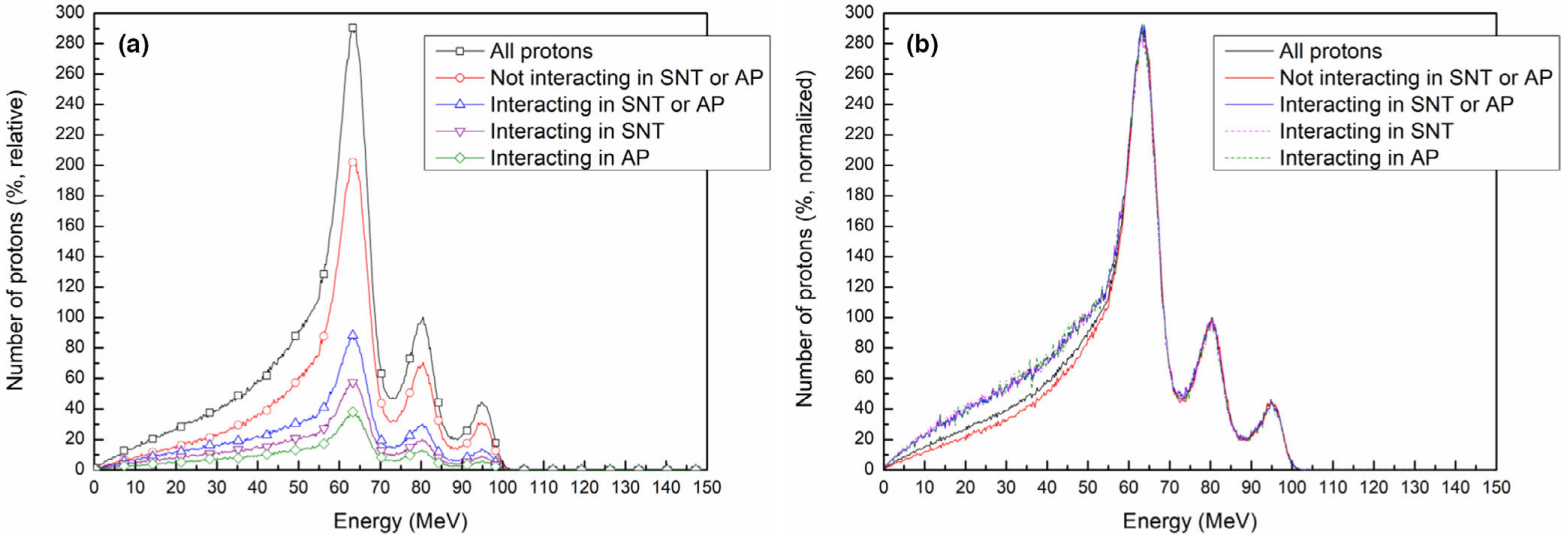
Effects of the applied filters in simulations on energy histograms of protons after passing through the range compensator: (a) the histograms on a relative scale, (b) the histograms normalized to the maximum of the second peak in each histogram.

Figure 8 shows the energy histograms of protons in the previous simulations on a relative scale (a) and a normalized scale (b). These energy histograms for classes of protons were acquired downstream of the range compensator. The three peaks in the energy histograms correspond to the energies of protons that passed through three different depths in the range compensator. In Fig. 8(a), the number of protons is normalized to the maximum of the second peak in the histogram of all protons, which corresponded to the center of the SOBP. Figure 8(b) shows the same histograms normalized to the maximum of the second peak in each histogram. As shown in the normalized histogram (Fig. 8(b)), the ratio of low‐energy protons to all protons was higher in the histogram of protons scattered from the snout or aperture than in the histogram of protons without scattering. These low‐energy protons cannot penetrate deep into the solid water phantom, and the contribution of scattered protons to the additional dose stands out only at shallow depths, as shown in the PDD profiles (Fig. 7).

For dose calculation in the TPS, which does not completely include the scattering effect, the relevant energy histogram is that for protons that do not interact in the snout and aperture, even this histogram includes a slight effect of scattering at the front parts of the nozzle. In reality, the addition of low‐energy protons from scattering to the ideal histogram of protons without interactions results in proton beams with energy distributions similar to the energy histogram for all protons. This difference between histograms may explain, at least in part, the dose differences of TPS calculations, Monte Carlo simulations, and measurements (Fig. 3, 4).

### 3.C Effects of snout sizes and beam ranges

**Fig. 9:**
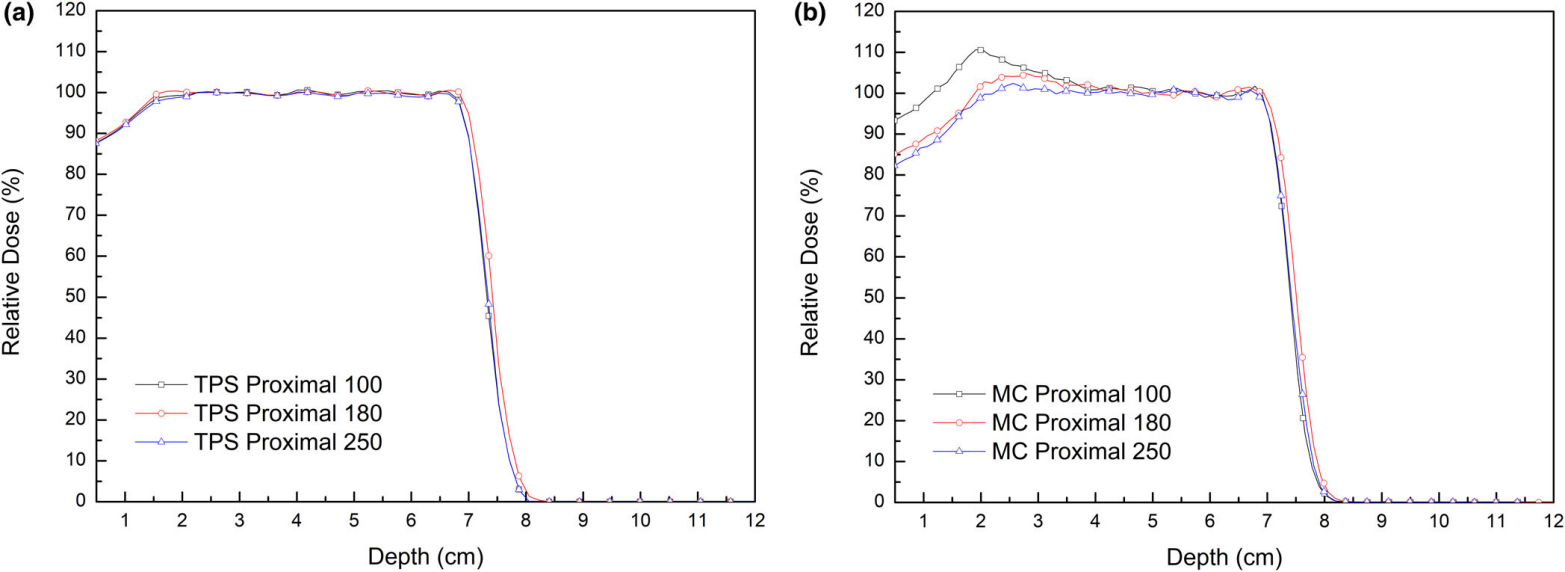
Effects of snout sizes on PDD profiles on CAX from TPS calculations (a) and Monte Carlo simulations (MC) (b) in the Proximal 100, Proximal 180 and Proximal 250 virtual treatment plans. Proton beams were of the same range.

The effects of snout sizes and beam ranges on proton scattering were investigated. Figure 9 shows the PDD profiles on CAX from TPS calculations (a) and Monte Carlo simulations (b) for virtual treatment plans using different sized snouts and proton beams of similar range (Table 3). All profiles were normalized to the dose at the center of the SOBP (a depth of 4.2 cm). Increasing the snout size resulted in a reduced dose at shallow depths for PDD profiles in the simulation, with the PDD profile approaching the ideal profile. In contrast, changing the snout size had little effect on PDD profiles calculated by the TPS. Increasing the inner diameter of the snout from 100 mm to 180 mm significantly reduced the proton dose at shallow depths, a dose mainly due to scattered protons, whereas a further increase in snout diameter from 180 mm to 250 mm slightly reduced the proton dose. This dose reduction may be due to greater spreading of the scattered protons from the snout and aperture into the wider field. Similar findings were observed when snout sizes were altered during the testing of cases with the same target as in the Proximal 100 plan.

**Fig. 10:**
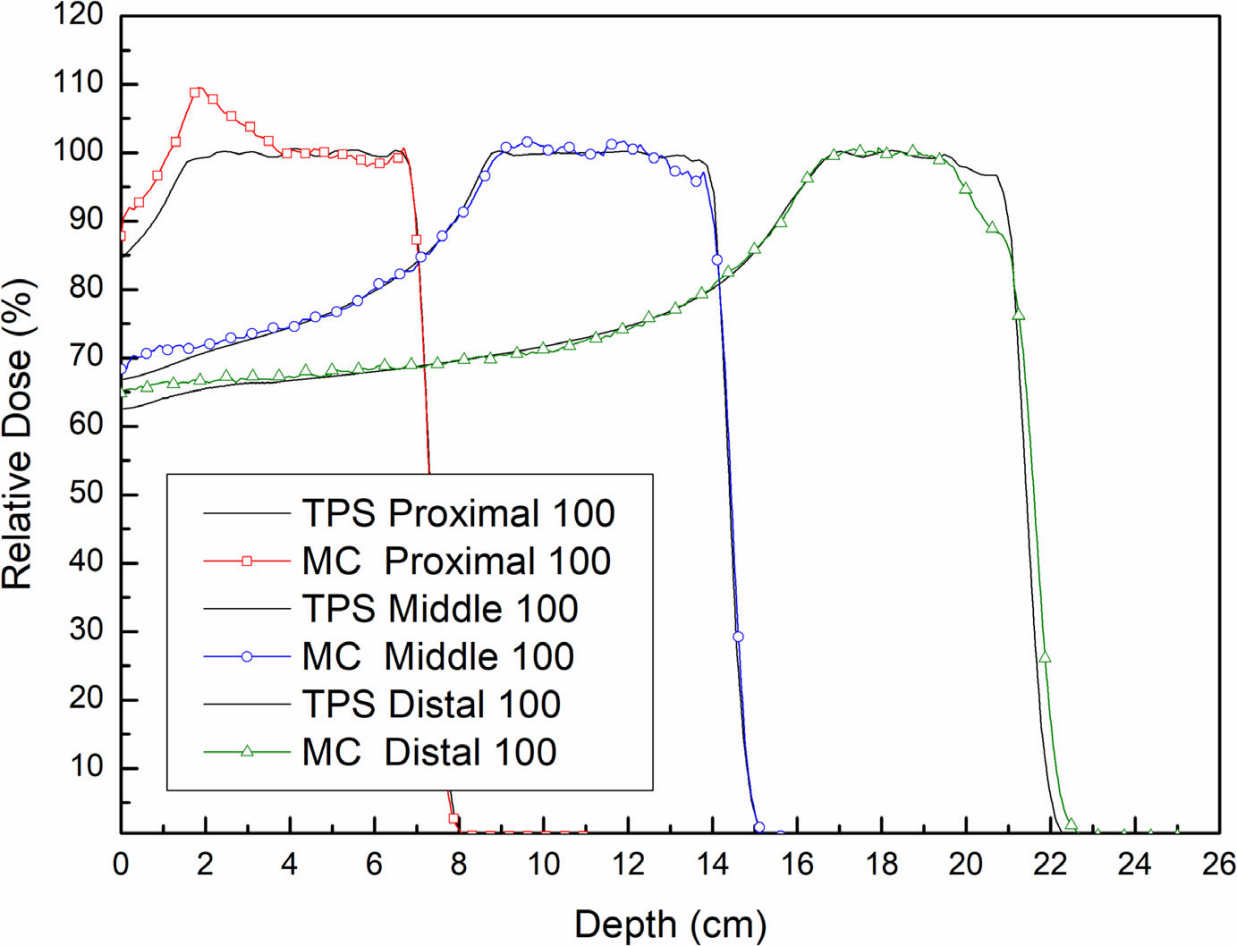
Effects of proton beam ranges on PDD profiles on CAX from TPS calculations and Monte Carlo (MC) simulations in the Proximal 100, Middle 100, and Distal 100 virtual treatment plans with the same snout size (SNT 100).

Moreover, the additional dose at shallow depths was found even when the proton beam range increased. Figure 10 shows the PDD profiles on CAX in TPS calculations and Monte Carlo simulations for virtual treatment plans with the same snout (SNT 100) but proton beams of different ranges (Table 3). All profiles were normalized to the dose at the center of the SOBP. Although increasing proton beam range resulted in a slight decrease in additional dose at shallow depths, the profiles from Monte Carlo simulations all included additional doses at depths of 40‐50 mm, confirming that the additional dose at shallow depths from scattered protons is not negligible for narrow snouts regardless of the beam range. The other noticeable difference was the collapse of SOBP in the distal region in the simulated profiles when the range increased. As the range increased, scattering in the range compensator has a greater effect on the proton beam divergence so that the deposited dose is more spread out laterally. This spreading of dose distribution can result in the lower dose in the narrow region at the bottom of dose distribution. Because these plans were established just for comparison of the dose in the proximal regions, the plan parameters were not optimized for each plan. It seems that enough smearing margin is required to the range compensator for covering the distal targets.

### 3.D Effects of the compensator base on the dosimetric effects of scattered protons

The effects of the compensator based on the effects of scattered protons were also analyzed in measurements and Monte Carlo simulations. Virtual treatment plans covering the same target volume in the Proximal 100 plan, but with different compensator bases, were used (Table 4). The resulting IDD profiles normalized to the dose at the center of the SOBP (a depth of 4.2 mm) were compared.

**Fig. 11:**
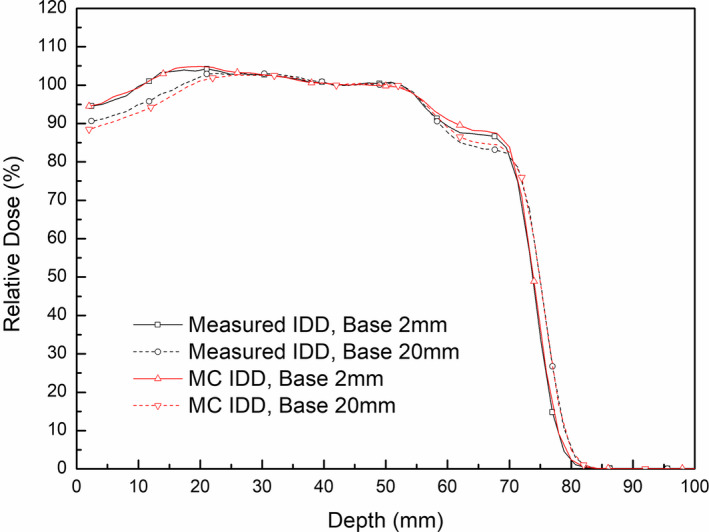
Effects of range compensator bases on IDD profiles in measurement and MC simulation.

Figure 11 shows comparisons of the simulated and measured IDD profiles of the plans with different compensator bases. The small active area of the multilayer ion chamber (2.5 cm in diameter) may not be appropriate to measure average doses under the thinnest region of a range compensator (2.25 cm in diameter, Fig. 6), and may cause the collapse in the distal region in both simulations and measurements. Even the collecting volume of ion chambers was considered in the simulation, there is a little difference between the simulated and measured profiles. But, despite this difference due to the approximation of the MLIC detector, both the measured and simulated results showed dose reductions at depths <2 cm following the application of the 20 mm range compensator base. In the simulations, compared with the dose at the center of the SOBP, the maximum dose differences with bases of 2 mm and 20 mm were 4.9% and 2.5%, respectively. Similarly, an increase in the thickness of the range compensator base reduced the maximum dose difference from 4.4% to 2.9% in the measurements.

**Fig. 12:**
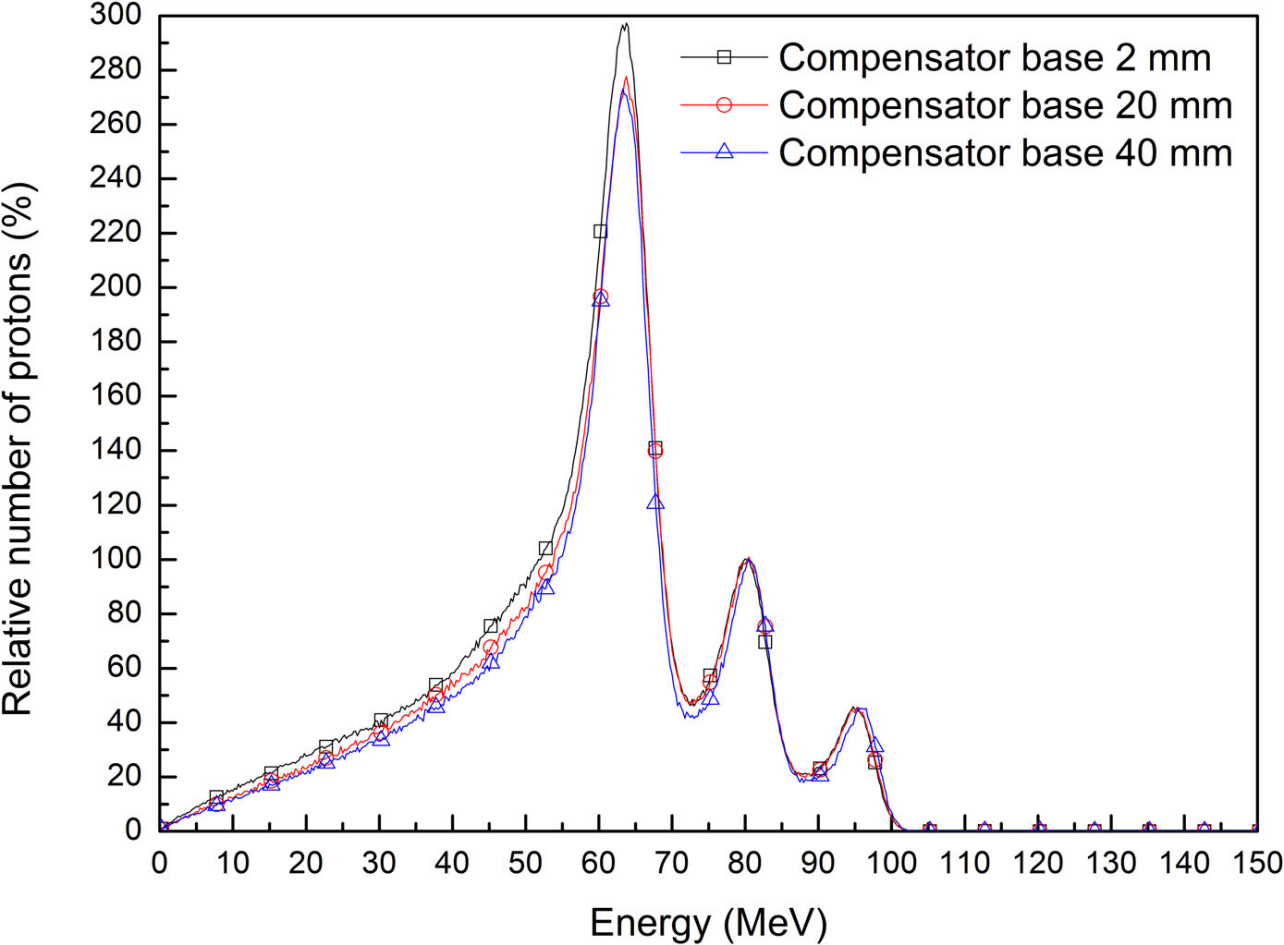
Energy histograms of protons after passing through a range compensator in the virtual plans with different compensator bases.

The effect of range compensators with thicker bases to reduce the dose from scattered protons can be explained by energy histograms of protons in Monte Carlo simulations. Figure 12 compares the energy histograms of all protons downstream of a range compensator in virtual plans with different base thicknesses. A histogram of protons with a range compensator base of 40 mm thickness, which is rarely used clinically, was included to investigate the dependence of base thickness. All histograms were normalized to the number of protons in the second peak of high energy, corresponding to the center of the SOBP. Increasing the base thickness of a range compensator reduces the number of low‐energy protons under 70 MeV, which contribute to the additional dose at shallow depths. This reduction in low‐energy protons was in agreement with the previously described the reduction of additional doses at shallow depths.

**Fig. 13:**
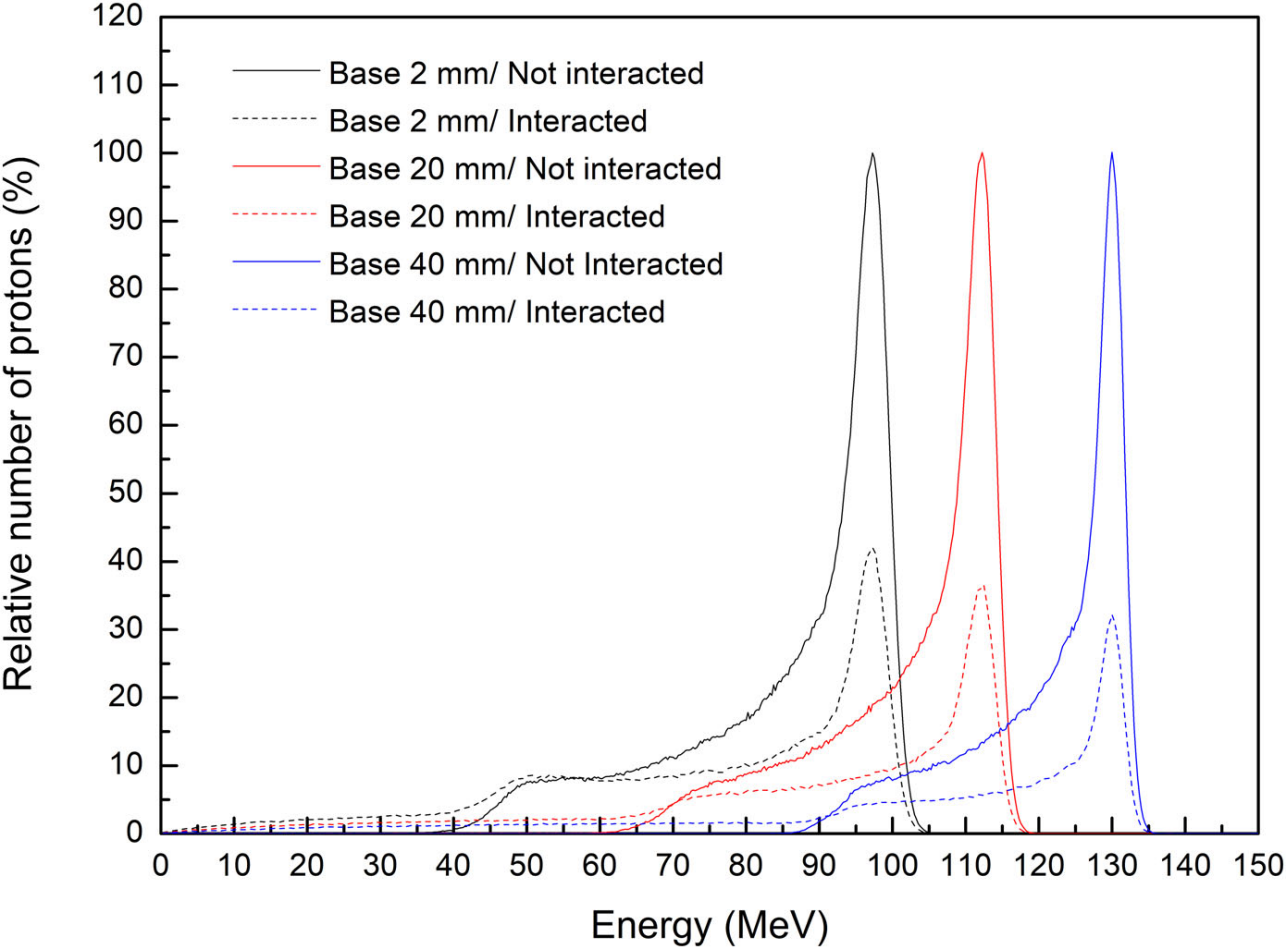
Effects of range compensator base thickness on energy histograms of scattered and passed protons without interactions after the aperture.

An additional analysis of energy histograms of protons before entering the range compensator can help understand the effect of a thicker compensator base on the additional dose. Figure 13 shows energy histograms of protons that were collected after passing the aperture with applied filters to scattering. These histograms were normalized to the maximum number of passed protons, enabling comparisons of the ratios of scattered to passed protons. The use of a thicker range compensator base reduced the ratio of scattered to passed protons without interactions at the snout and aperture when the same dose was delivered. This relationship between reduced scattering and increased base thickness of the range compensator can be understood by evaluating the relationship between the energy and directions of proton beams. An increase in base thickness of a range compensator increased the range of the proton beam to cover the same target volume. When the proton beam is of higher energy, the direction of the beam is intensified in a forward direction, reducing scattering at the snout and aperture.

## 4 DISCUSSION

The dosimetric effect of protons scattered from the snout and aperture passive scattering proton therapy was measured and evaluated in clinical practice when a range compensator was included. The results of this study confirmed previous results based on Monte Carlo simulations,^9^, ^13^ and showed that the effect of scattered protons can persist even after passing through a range compensator. These analyses indicated that the dosimetric effect of scattered protons is more complicated than previously thought, and requires more detailed consideration in practice.

The snout used to insert the aperture and the range compensator has another role to protect patients from unwanted radiation. The structure and material composition of the snout vary by supplier and site, but it is usually composed of high‐Z materials. The snouts can make a non‐negligible contribution to proton scattering, depending on their design. The combination of a large aperture opening and a narrow snout can be considered an extended aperture. Increases in aperture length have been associated with increases in the dose from scattered protons.^13^ In this study, the use of a long and narrow snout, 30 cm in length and 11 cm in diameter, in combination with an aperture with a wide opening, resulted in protons scattered from the snout having a more dominant effect than protons scattered from the aperture. This result indicates that the use of a snout always results in scattered protons, and that the dosimetric effects of these scattered protons are not limited to small fields <5 cm in diameter.

The range compensator for distal shaping of the beam also affects the dosimetric contribution of scattered protons. The direction of scattered protons can spread out over large angles differently from the beam direction on TPS. Scattered protons that enter thick areas of the range compensator are of low energy and are therefore absorbed in the superficial region of the phantom. Only scattered protons that pass through thin areas of the range compensator can result in considerable doses at shallow depths. Consequently, the dosimetric effect of the scattered protons is filtered out by the geometric structure of the range compensator.

A comparison with the dose calculated by the TPS showed that considering the potential dose variation by scattered protons is required in treatment planning. Especially when the full‐modulation SOBP beam is used to cover the superficial region, an unexpected additional dose can be critical in the superficial region. In clinical practice, the number of beams usable is limited in passive scattering proton therapy, making it difficult to reduce the dose to the superficial region only by splitting proton beams in several directions.

Introduction of the effect by scattered protons into the dose calculation engine of the TPS is the ultimate solution for this issue. The previous study, which added the approximated model of the contribution of aperture‐scattered protons in analytical dose calculation, demonstrated a reduction of discrepancies between TPS calculation and measurement.^16^ Nevertheless, the extension of the analytical dose calculation algorithms to include the contribution of the scattered protons is difficult if the effects of the snout and range compensator are considered together. The Monte Carlo‐based TPS can reduce this dose variation by including scattering at the snout and aperture in calculation, even without the full MC simulation. But it requires the increased calculation time in this case, and the current Monte Carlo‐based TPS are not developed for the conventional scattering proton therapy.

Additional investigation of the dosimetric effects of snout size, beam range, and range compensator base thickness can provide more options applicable to the current commercial planning systems. The present study showed that the use of a wider snout leads to the spreading of scattered protons, and that use of a thicker compensator base leads to a reduction in the number of scattered protons. Both methods can reduce the additional dose provided by scattered protons while delivering the desired dose to the target. These methods can complement the use of multiple beams in reducing additional doses in clinical practice.

The limitation of this study is the exclusion of the dose by the secondary neutrons. It is well known that the snout and the aperture is the predominant source for neutron dose to patients in the passive scattering proton therapy.^25^ The dose deposited by the secondary neutrons is not negligible because of their higher biological effectiveness. Because neutrons show different attenuation characteristics with depth from that of protons and are less affected by the range compensator, this study was focused on the dosimetric effect of the scattered protons and did not include the effect of the secondary neutrons. The dosimetric effect of secondary neutrons during the passive scattering proton therapy can be referred to in the previous studies.^26^, ^27^

## 5 CONCLUSION

The dosimetric effect of scattered protons in passive scattering proton therapy, as previously investigated by Monte Carlo simulation, was verified in the present study by measurement and analysis in clinical practice when a range compensator was included. This dosimetric effect at shallow depths was observed even when a range compensator was included, although the dosimetric effect was filtered by the structure of the compensator. Protons scattered from the snout provided a significantly greater contribution to dose than protons scattered from the aperture, especially with the snout for small fields. These results indicate that potential dose variations at shallow depths should be considered in treatment planning in cases using narrow snouts. Studies investigating the effects of snout size, beam range, and range compensator base thickness on the effect of scattered protons can help design methods to reduce the dosimetric effects of scattered protons, even when using commercial TPS.

## Data Availability

The data that support the findings of this study are openly available in [Default Parameters] at [https://topas.readthedocs.io/en/latest/], reference number [23].
